# Diversifying and correlational selection on behavior toward conspecific and heterospecific competitors in brook stickleback (*Culaea inconstans*)

**DOI:** 10.1002/ece3.339

**Published:** 2012-07-25

**Authors:** Kathryn S Peiman, Beren W Robinson

**Affiliations:** Department of Integrative Biology, University of GuelphGuelph, Ontario, Canada

**Keywords:** Adaptive behavior, behavioral syndrome, direct interactions, interspecific agonism, species recognition

## Abstract

Behaviors toward heterospecifics and conspecifics may be correlated because of shared mechanisms of expression in both social contexts (nonadaptive covariation) or because correlational selection favors adaptive covariation. We evaluated these hypotheses by comparing behavior toward conspecifics and heterospecifics in brook stickleback (*Culaea inconstans*) from three populations sympatric with and three allopatric from a competitor, the ninespine stickleback (*Pungitius pungitius*). Behavioral traits were classified into three multivariate components: overt aggression, sociability, and activity. The correlation of behavior between social contexts for both overt aggression and activity varied among populations in a way unrelated to sympatry with ninespine stickleback, while mean aggression was reduced in sympatry. Correlations in allopatric populations suggest that overt aggression and activity may genetically covary between social contexts for nonadaptive reasons. Sociability was rarely correlated in allopatry but was consistently correlated in sympatry despite reduced mean sociability, suggesting that correlational selection may favor a sociability syndrome in brook stickleback when they coexist with ninespine stickleback. Thus, interspecific competition may impose diversifying selection on behavior among populations, although the causes of correlated behavior toward conspecifics and heterospecifics and whether it can evolve in one social context independent of the other may depend on the type of behavior.

## Introduction

Aggressive behavior is prevalent in animals and is usually context dependent, as is our understanding of its causes and consequences. For example, the proximal and ultimate functions of aggression, such as establishing dominance or eliciting submissive behavior, have been studied in conspecific contexts but are less understood during heterospecific interactions (Parker 1974; Grether et al. 2009; Ord and Stamps 2009; Wilson et al. 2009; Peiman and Robinson 2010; Wade et al. 2010; Ord et al. 2011). Conspecific aggression (CA) regularly influences fitness under competition for resources and mates (Elliott 1986; Lahti et al. 2001) and can generate social selection (West-Eberhard 1979; Bleakley et al. 2007; Bleakley and Brodie 2009; McGlothlin et al. 2010). Heritable variation in CA is found in many taxa, including mammals (Hall and Klein 1942; D'Eath et al. 2009; Wilson et al. 2009; Taylor et al. 2011), birds (Fennell 1945), fish (Bakker 1986), and insects (Hoffmann 1988; Edwards et al. 2006), although heritability can also depend on context (Wilson et al. 2009). CA can be correlated with behaviors in other contexts (forming behavioral syndromes; e.g., Conrad et al. 2011), such as boldness toward predators (Huntingford 1976a, 1982; Bell and Stamps 2004; Bell 2005; Bell and Sih 2007; Dochtermann and Jenkins 2007) and activity in unfamiliar environments (Huntingford 1976a; Verbeek et al. 1996; Bell and Stamps 2004; Bell 2005; Dingemanse et al. 2007; Kortet and Hedrick 2007). CA can also have ecological effects, such as influencing predation risk (Baker et al. 1998), contributing to dispersal (Duckworth 2008), and affecting the spatial distribution of individuals through territoriality (Maher and Lott 1995). Thus, the ecological and evolutionary effects of aggression have been fairly well studied in conspecific contexts.

Heterospecific aggression has been generally regarded as less important than aggression toward conspecifics with respect to its ecological and evolutionary consequences (Peiman and Robinson 2010). Heterospecific aggression (HA) may evolve under selection arising from resource competition (Peiman and Robinson 2010) because HA can affect fitness (Downes and Bauwens 2002; Eccard and Ylonen 2002; Duckworth 2006) and its variation appears heritable (Duckworth and Badyaev 2007; Peiman and Robinson 2007). One potentially important ecological consequence of HA is the possible local extirpation of native species by aggressive invaders (Holway and Suarez 1999).

Many studies appear to assume that aggression is correlated between conspecific and heterospecific contexts because HA is often scaled against CA. This assumption seems reasonable because the functions and structures or mechanisms of expressing aggression appear to be largely similar in both social contexts (hereafter “social context” refers to interactions with conspecifics vs. with heterospecifics). Functionally, aggression in either social context can secure access to defendable resources such as food, shelter, and breeding sites, while mates are a uniquely conspecific resource. The structures and mechanisms used to express aggression may also be the same in both social contexts. These include morphological weapons (mouth, limbs, claws), signals (coloration, vocalization, displays), and physiological, hormonal, and neural-muscular regulatory systems (Vowles and Harwood 1966; Bakker 1994; reviewed in Huntingford 1976b). Strongly shared structures and functions may cause HA to positively covary with CA. However, a recent review of 273 tests (Peiman and Robinson 2010) found only two (Huntingford 1976a; Duckworth 2006) that had tested this assumption, and both found that HA positively covaried with CA among individuals. All other studies have only estimated population mean levels of CA and HA (Clark and Ehlinger 1987; Wilson 1998; Bolnick et al. 2003; Dall et al. 2004), and so the extent to which HA covaries with CA among individuals or populations is largely unknown for most animals.

The covariation of CA with HA may occur for two reasons. CA may covary with HA because of shared mechanisms under genetic control, which we refer to as the nonadaptive genetic covariation hypothesis because the correlation of behavior between social contexts has not evolved under selection arising from interspecific and intraspecific interactions. Genetic correlations can potentially constrain the evolution of aggression as it cannot independently evolve in each social context (Lande 1979; Lande and Arnold 1983; Ketterson and Nolan 1999; Bell 2005; Kirkpatrick 2009). However, theory suggests that the same behavior expressed in different contexts can be controlled by different genes (Reale et al. 2007) which may allow aggression to evolve under selection in one context independent of the other or to adaptively covary, depending on the nature of the underlying adaptive landscape and preexisting genetic architecture (Dingemanse et al. 2010). If the effects of CA and HA on fitness are independent, then selection acting on CA and/or HA will favor a single set of CA–HA values that represent the local fitness peak and no adaptive correlation will evolve in the population. If the effects of CA and HA interact to jointly determine fitness and more than one pair of CA–HA values are of equal fitness, then selection on aggression is correlational and the population evolves on a fitness ridge (Dingemanse and Reale 2005). Here, adaptive covariation may evolve if personality types expressing low CA and low HA and those expressing high levels of both have equally high fitness. A genetic correlation between the traits may then evolve (Bell and Sih 2007; Dingemanse et al. 2007, 2009) or not, depending on available genetic variation (Sinervo and Svensson 2002).

Comparative approaches like we employ here can test hypotheses about how selection and constraints may influence the evolution of behavior. Stabilizing or directional selection acting within populations is typically evaluated using mean phenotypic values (e.g., Foster and Endler 1999), while correlational selection is evaluated using the strength and/or direction of phenotypic correlations within populations. If behavior expressed in different contexts can be controlled by different genes, then diversifying selection should result in phenotypes (means or correlations) varying among evolutionarily independent populations under different local ecological conditions, but remaining consistent among those populations sharing a common selective environment (Dingemanse et al. 2007). This requires *a priori* identification of hypothetically divergent selection acting among populations (Dochtermann 2010) and the replication of populations in divergent environments in order to distinguish neutral from adaptive evolution (Harvey and Pagel 1991). In contrast, if CA and HA strongly genetically covary, then HA will be positively correlated with CA within replicated populations regardless of variation in local environmental conditions. In addition, changes in mean aggression in one social context should cause mean aggression to change in the other context, so that mean values of HA and CA will positively covary among populations (Lande 1979; Bell 2005; Dingemanse and Reale 2005).

**Figure 1:**
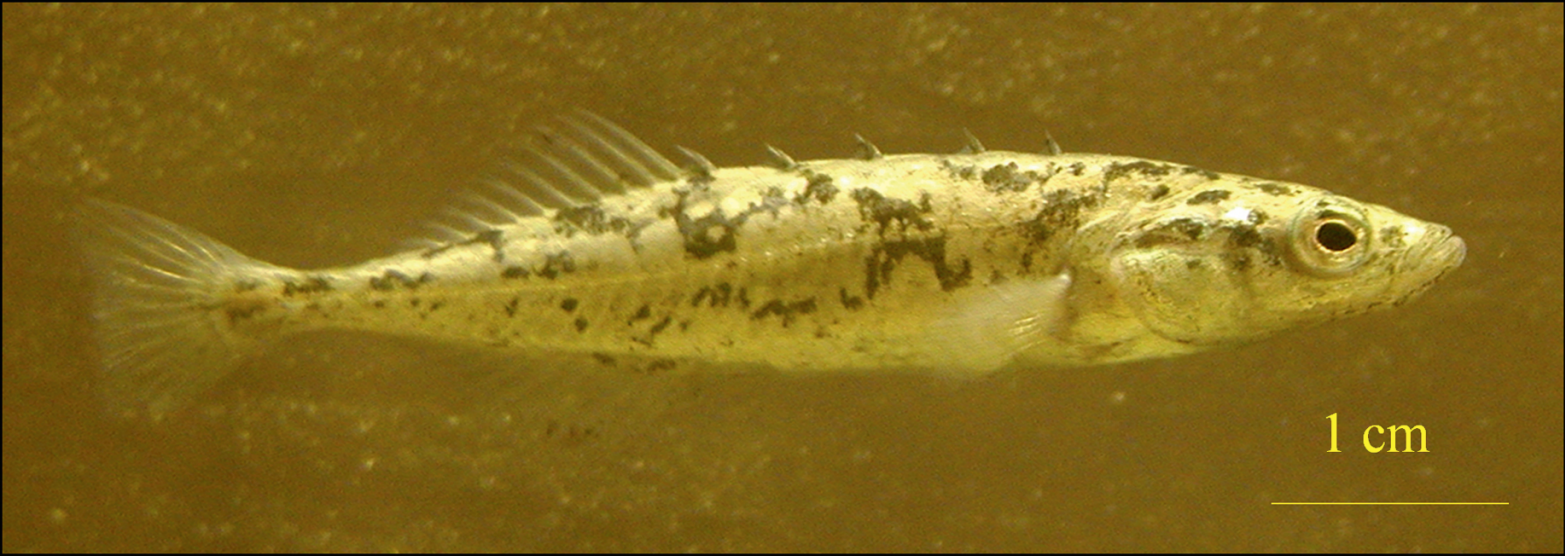
Brook stickleback (*Culaea inconstans*).

Phenotypic changes in mean HA independent of CA have been observed in a variety of taxa by comparing aggression between populations sympatric with and allopatric from a competitor (Peiman and Robinson 2010), and numerous studies have also examined behavioral covariation among populations that vary with respect to predator presence (i.e., Brown et al. 2005; Alvarez and Bell 2007). However, no studies have yet focused on the relationship between aggressive behaviors toward conspecific and heterospecific competitors among populations that vary in the presence of the competitor. Our objective was to estimate this relationship in brook stickleback (*Culaea inconstans*; Fig 1), and by using variation among populations, to evaluate how behavior may have evolved. Populations of brook stickleback are found both allopatric from and sympatric with ninespine stickleback (*Pungitius pungitius*). Both species share a similar ecological niche in the shallow inshore waters (Wootton 1976; Gray et al. 2005) where interspecific competition for defendable benthic resources affects growth and morphology (Gray and Robinson 2002; Gray et al. 2005). Adult wild-caught sympatric brook stickleback were more aggressive toward ninespine stickleback than allopatric brook stickleback, suggesting that HA is beneficial in the presence of a competitor (Peiman and Robinson 2007). Variation in HA was also heritable, as juvenile brook stickleback from sympatric populations were more aggressive toward ninespine stickleback than allopatric brook stickleback when reared in a common laboratory environment (Peiman and Robinson 2007).

Here, we evaluate the extent to which HA phenotypically covaries with CA and explore the potential evolutionary implications of such covariation. If correlational selection arises from competition between brook and ninespine stickleback in sympatry, then sympatric populations of brook stickleback will exhibit stronger phenotypic correlations between HA and CA than allopatric populations. Alternatively, if HA is strongly genetically correlated with CA, then HA and CA will be positively correlated in all brook stickleback populations (including allopatric populations where brook stickleback are naive with respect to ninespine stickleback), and population mean HA and CA will also covary among populations.

## Methods

### Stickleback collection

Fieldwork was conducted at Esker Lakes Provincial Park in north central Ontario (Canada), which contains numerous small, isolated, and oligotrophic kettle lakes. The fish assemblages are generally depauperate and are slightly less diverse in allopatric lakes than in sympatric lakes (Table 1). Allopatric brook stickleback from three lakes (Bea, Armitage, and Dewhirst) and sympatric brook stickleback from three lakes (Rozon, Lallan, and Garrison) used in prior studies (Gray and Robinson 2002; Gray et al. 2005; Peiman and Robinson 2007) were collected using standard minnow traps in August 2008, concurrent with behavioral trials. Brook stickleback density varied among populations and was highest in Armitage, Garrison, and Rozon (0.74–0.81 brooks/trap hour) and lower in Bea, Dewhirst, and Lallan (0.18–0.27 brooks/trap hour). Garrison had approximately three times as many ninespine stickleback as Rozon, and the density of ninespine stickleback was very low in Lallan (different capture methods precludes comparing catch per unit effort estimates between species).

**Table 1:** Characteristics of kettle lakes (mean ± SEM) used in this and prior studies (from Gray 2001)

	Sympatric	Allopatric
Fetch (km)	0.40 ± 0.15	0.54 ± 0.13
Maximum depth (m)	19.9 ± 8.0	11.6 ± 6.9
Secchi depth (m)	5.8 ± 1.5	5.0 ± 1.3
Fish fauna		
*Culaea inconstans*	Brook stickleback	Brook stickleback
*Salvelinus fontinalis*	Brook charr	Brook charr
*Chrosomus eos* and *C. neogaeus*	Dace	Dace
*Pungitius pungitius*	Ninespine stickleback	
*Semotilus atromaculatus*	Creek chub	
*Notropis volucellus* and *N. hudsonius*	Shiners	
*Catostomus commersonii*	White sucker	
*Cottus* spp.	Sculpins	

### Quantifying behavior

Individual brook stickleback (hereafter “residents”) were housed indoors in 18-L opaque plastic tubs (33 cm long × 22 cm wide × 22 cm deep) with sand substrate. A small plastic plant was placed at one end of each tub as a refuge. A window down one long side allowed observation of the resident during interactions with a contained intruder placed at the other end. Water temperature varied with ambient air temperature from 14 to 20°C, and lighting consisted of ceiling bulbs on a 14-h light:10-h dark cycle. Residents were fed bloodworms each evening.

Healthy adults were selected as the resident regardless of body coloration or reproductive state. Nuptial coloration occurred in males in less than 5% of trials, and females were never obviously gravid. Single residents were introduced to each tub each evening. Testing with an intruder began the following morning. Intruders were healthy, but not nuptially colored or obviously gravid. Single intruders were presented in a clear acrylic cylinder (6 cm diameter × 25.7 cm high) in the same location food had been presented. Each resident was sequentially presented with a brook and ninespine stickleback intruder in random order (hereafter called intruder order), with at least 4 h between trials. Both intruder species came from the same sympatric lake that was not the resident's lake, requiring two brook and ninespine intruders for each replicate of six lakes. Individuals from all six populations were tested concurrently. The experimenter was blind to resident population origin. All sticklebacks were euthanized after trials to obtain standard length and determine sex. All procedures were reviewed and approved by the University of Guelph's Animal Care Committee.

Resident brook stickleback behavior was quantified for each valid trial. A trial was considered valid when the resident approached within 5 cm of the intruder within 10 min of its presentation. Resident behavior was recorded for an additional 10 min after first approach in real time using JWatcher 0.9 (Blumstein et al. 2000): (1) Latency: the time the resident took from initial presentation of the intruder to approach within 5 cm; (2) Bite: a discrete event consisting of an open-mouth contact with the cylinder directed at the intruder; (3) Bout: a continuous series of head bumps against the cylinder directed at the intruder, ending when the resident broke contact with the cylinder for >1 sec; (4) Number of visits: the number of times the resident crossed the centerline into the intruder half of the tub; (5) Broadside threat: a discrete event involving the head of the resident directed at the intruder while the body is held perpendicular to the intruder; (6) Number of orients: the number of times the resident turned to face the intruder; and (7 and 8) Two measurements of duration of orients: the total time oriented toward and total time oriented away from the intruder while in the intruder half of the tub, which are not reciprocal because they depend on the fraction of total trial time that a resident spends in the intruder half of the tub.

### Statistical analyses

Raw behavior variables were transformed to their square root (count + 3/8) to normalize distributions. We summarized the conspecific and heterospecific behavior of all individuals that responded to both species of intruders from all six populations using a full correlation-based principal component analyses (PCA). Individuals that only responded to one species of intruder were excluded because preliminary analysis showed that they had different scores than individuals responding to both intruders on PC2 (both > one) and PC3 (one > both), although loadings and correlation matrices were quite similar in both PCAs. We also analyzed behavior toward conspecifics and heterospecifics in separate PCAs and found that correlation matrices were similar for all principal components (data not shown), justifying combining behavior toward both species of intruder. We also investigated variation in HA over time between this data set (from 2008) and these same populations assessed in 2004 (see Peiman and Robinson 2007) by constructing a multiyear PCA of only HA from allopatric and sympatric individuals used in the final analyses in 2004 and 2008 (variation in CA could not be investigated because it was not assessed in 2004).

The first three components of the full PCA had eigenvalues greater than one, and so were retained. This generated scores on six multivariate components for each individual: conspecific behavior PC1–PC3 and heterospecific behavior PC1–PC3. We initially used a mixed effects hierarchical MANOVA (multivariate analysis of variance) model to test the effects of sympatry/allopatry and intruder species on behavioral variation in PC1–PC3. The sym/allo effect was tested against variation among populations nested in sym/allo; lake effects were tested against variation among individuals nested in populations. We then performed separate analyses of variation in each PC using the same nested model to focus on particular behaviors. We could not include all other effects in a single model because our data were either seriously unbalanced across these additional factors (relative size – whether the resident was larger or smaller than the intruder, resident sex, and intruder sex), or because individuals had no variation in that effect (intruder order). We explored the effects of these additional factors using various modified models and found evidence that some of these factors influenced behavioral variation though often in complex ways (results not shown), and so will contribute to residual variation in our models above.

Spearman rank correlation coefficients between conspecific and heterospecific behaviors were then estimated for each PC within each population. A behavioral syndrome is defined by between-individual consistency in rank order (Sih et al. 2004a,b) and so ranks are often used in studies of behavioral covariation (e.g., Bell 2005; Dingemanse et al. 2007). Ninety-five percent confidence intervals for the mean correlation of populations in sympatry versus allopatry were determined on Z-transformed coefficients for each PC.

We also evaluated whether the absence of correlations was related to low among-individual variation in conspecific or heterospecific scores using Wilcoxon two-sample tests (six tests total; one-sided alternate hypotheses of variance in correlated populations > uncorrelated populations for PC1–PC3). Additionally, we used Wilcoxon two-sample tests to determine if the variance of conspecific and heterospecific scores on PC1–PC3 differed between sympatric and allopatric populations (six tests total) treating population as the independent unit. Lastly, we tested for differences in variation between conspecific and heterospecific scores for each PC and population using Levene's tests (18 tests in total) treating individuals as the independent unit. All tests were two-sided except where indicated and were performed using JMPin (SAS Institute, Inc, Cary, North Carolina).

## Results

### Within-year comparison of CA and HA

Of 332 brook stickleback tested, 150 (68 allopatric and 82 sympatric) provided valid conspecific and heterospecific trials, while an additional 37 had valid conspecific trials only (13 allopatric, 24 sympatric) and 37 had valid heterospecific trials only (17 allopatric, 20 sympatric). Five populations had a high percentage of individuals with at least one valid trial (Armitage: 73.3%, Bea: 80.5%, Dewhirst: 91.4%, Lallan: 85.5%, Garrison: 60%), while Rozon had the fewest responders (37.8%). The mean behaviors of fish from Rozon were within the range of the other five populations (Table 2). The proportion of nonrespondents did not vary between allopatric and sympatric populations (Chi-square test: χ^2^ = 1.2, *P* = 0.28). Residents tended to respond to both or to neither intruder (Chi-square test: χ^2^ = 104.2, *P* < 0.0001).

**Table 2:** Population means (and SEM) of behaviors for brook stickleback residents toward conspecific (C) and heterospecific (H; ninespine stickleback) intruders. Armitage, Bea, and Dewhirst are allopatric from ninespine stickleback; Garrison, Lallan, and Rozon are sympatric with ninespine stickleback

Behavior	Intruder species	Armitage	Bea	Dewhirst	Garrison	Lallan	Rozon
Number of times oriented toward intruder	C	20.8 (1.9)	13.1 (1.8)	18.9 (2.2)	18.7 (2.4)	15.6 (1.6)	17.8 (2.6)
	H	21.0 (1.5)	18.6 (1.9)	20.3 (2.5)	15.0 (2.1)	18.5 (1.4)	18.1 (2.6)
Total time oriented toward intruder	C	314.8 (27.2)	411.1 (21.4)	406.3 (23.7)	285.4 (20.6)	311.9 (21.3)	321.7 (28.9)
	H	305.4 (22.6)	362.6 (24.3)	288.0 (27.5)	237.8 (26.0)	243.7 (20.3)	256.6 (35.0)
Total time oriented away from intruder	C	157.4 (16.7)	135.8 (15.6)	162.2 (19.9)	204.1 (21.8)	179.1 (16.6)	203.4 (29.1)
	H	179.2 (20.4)	164.2 (15.3)	223.8 (23.6)	210.1 (23.3)	212.2 (18.6)	228.0 (30.6)
Number of times visiting intruder	C	6.1 (1.4)	2.5 (0.4)	3.0 (0.6)	4.2 (0.5)	4.6 (0.6)	2.8 (0.3)
	H	6.5 (1.1)	3.9 (0.6)	4.1 (0.7)	4.0 (0.7)	6.4 (0.9)	2.7 (0.5)
Bite	C	20.1 (5.3)	32.6 (5.6)	27.1 (6.4)	14.0 (3.9)	7.9 (1.7)	18.4 (3.7)
	H	18.8 (6.2)	24.5 (4.8)	14.6 (5.2)	11.9 (3.1)	7.1 (1.4)	13.9 (4.9)
Threat	C	4.4 (1.1)	4.6 (1.1)	5.2 (1.4)	1.9 (0.6)	3.9 (1.0)	4.2 (1.4)
	H	5.2 (1.3)	2.1 (0.8)	2.9 (1.0)	0.5 (0.2)	1.9 (0.5)	1.7 (1.4)
Bout	C	4.8 (1.8)	8.0 (2.0)	4.5 (1.8)	6.3 (1.7)	1.9 (0.4)	6.8 (1.7)
	H	4.5 (1.4)	9.7 (2.7)	1.6 (0.9)	2.7 (1.0)	2.7 (0.8)	2.6 (1.1)
Latency	C	130.8 (30.2)	156.0 (31.5)	108.1 (20.5)	188.5 (30.8)	141.7 (20.4)	271.2 (39.0)
	H	211.5 (40.5)	97.3 (18.0)	99.0 (22.4)	216.3 (31.8)	143.6 (23.6)	237.1 (42.6)

The first three components of the full PCA captured 70% of the total variation in behavior (Table 3). PC1 captured 33% of variation, and positive scores reflected more time oriented toward the intruder, more bites, threats, and bouts. Following Reale et al.'s (2007) descriptions, we refer to PC1 as “aggression” toward the intruder. PC2 captured an additional 22% of variation, with positive scores reflecting more frequent orientation toward the intruder but also more time oriented away from the intruder, and negative scores reflecting increased latency. We refer to PC2 as “sociability” toward the intruder, as social individuals spend more time in the presence of the intruder (Dingemanse et al. 2009; Conrad et al. 2011). PC3 captured 15% of variation, and high scores reflected a higher frequency of movement across the tank to visit the intruder, which suggests “activity”, although here measured in a social situation.

**Table 3:** The canonical loadings of brook stickleback (C*ulaea inconstans*) behavior toward conspecific and heterospecific intruders. Absolute values greater than 0.4 are bolded to highlight their major contribution to behavioral variation on that component

Behavior	Overt aggression (PC1)	Sociability (PC2)	Activity (PC3)
Number of times oriented toward intruder	−0.06	**0.68**	0.23
Total time oriented toward intruder	**0.48**	0.21	−0.26
Total time oriented away from intruder	−0.3	**0.52**	−0.25
Number of times visiting intruder	−0.19	0.06	**0.81**
Bite	**0.43**	0.2	0.31
Threat	**0.43**	−0.13	0.16
Bout	**0.48**	0.02	0.1
Latency	−0.19	**−0.40**	0.18
Eigenvalue	2.62	1.73	1.20
Percent variance	32.7	21.6	15

**Figure 2:**
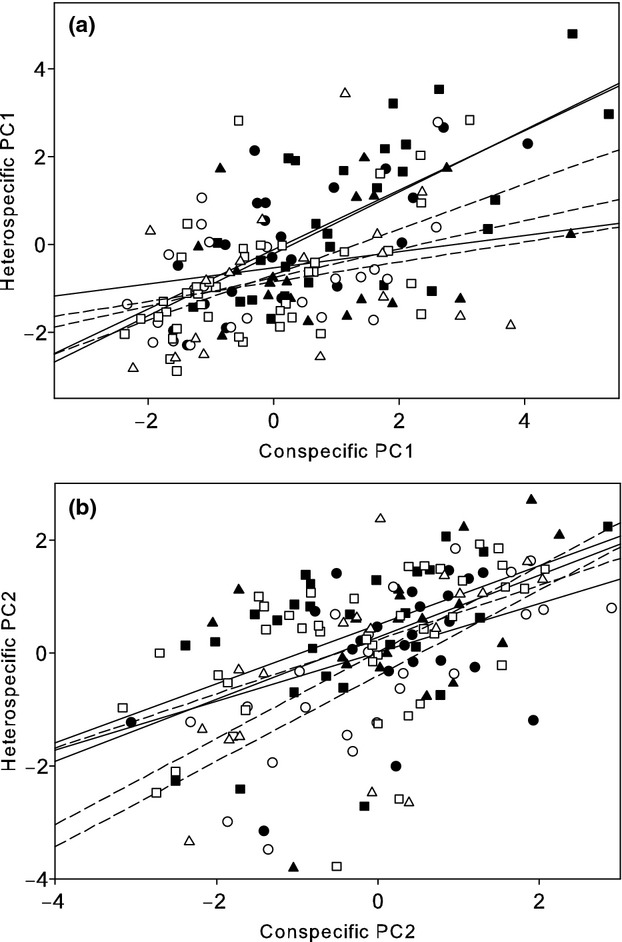
Behavior of brook stickleback (C*ulaea inconstans*) toward conspecifics and heterospecifics (ninespine stickleback, *Pungitius pungitius*) from three allopatric (Armitage: • Bea: ▪ Dewhirst: ▴; solid lines) and three sympatric (Garrison: ○ Lallan: □ Rozon: Δ; dashed lines) populations. (a) Variation in overt aggression (PC1), where positive scores reflect more overt aggression by the resident toward the intruder. Rank correlations between conspecific and heterospecific aggression were significant for Armitage, Bea, Garrison, and Lallan (see Table 5). (b) Variation in sociability (PC2), where positive scores reflect greater sociability with the intruder. Rank correlations between conspecific and heterospecific sociability were significant for Bea, Garrison, Lallan, and Rozon.

**Figure 3:**
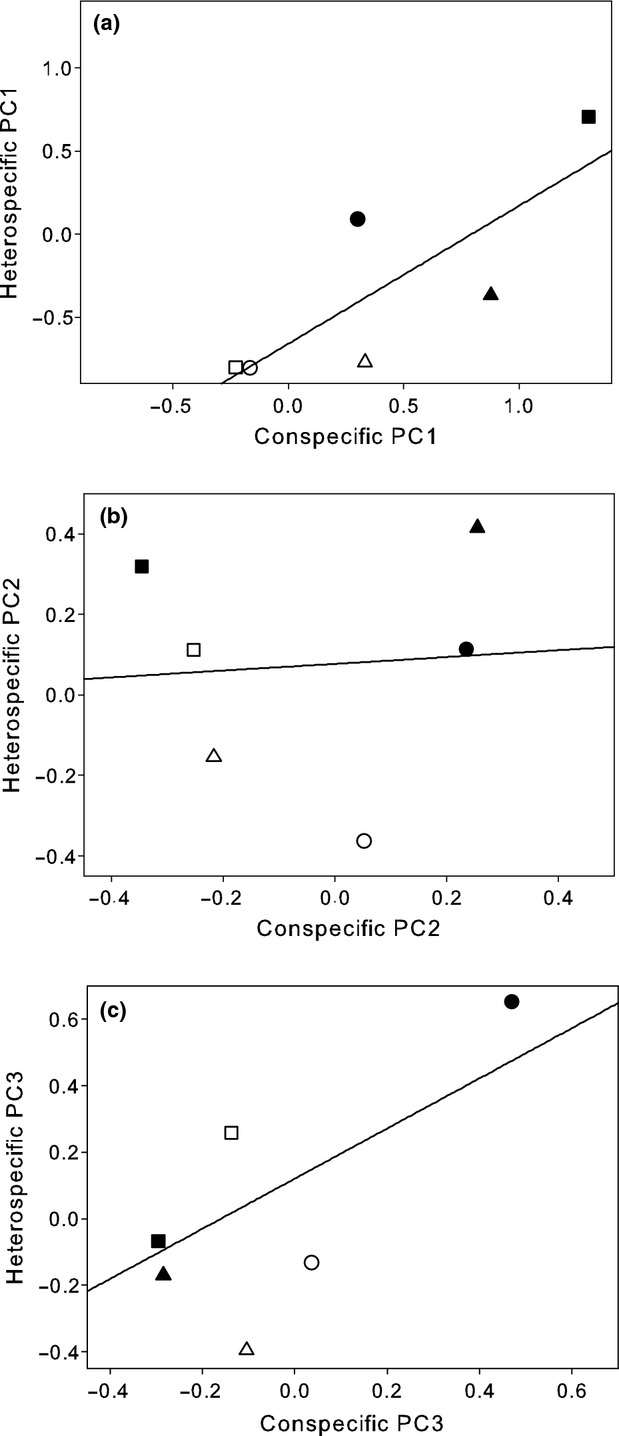
Mean behavior of brook stickleback (*Culaea* inconstans) behavior toward conspecifics and heterospecifics (ninespine stickleback, *Pungitius pungitius*) for three allopatric (Armitage: • Bea: ▪ Dewhirst: ▴; solid lines) and three sympatric (Garrison: ○ Lallan: □ Rozon: Δ; dashed lines) populations. Among-population mean values (a) were correlated for aggression (Pearson's *r* = 0.80, *P* = 0.055); (b) were not correlated for sociability (Pearson's *r* = 0.075, *P* = 0.89); (c) showed a positive trend for activity (Pearson's *r* = 0.72, *P* = 0.10).

Mean behavior varied with sympatry/allopatry, population, and intruder species (Table 4). Sympatric populations had lower mean behavior toward intruders than allopatric populations (MANOVA PC1–PC3; *P* = 0.001). Fish from all populations exhibited greater overt aggression toward conspecifics than heterospecifics (Fig 2a). Sympatric populations had lower mean aggression (Fig 2a) and sociability (Fig 2b) than allopatric populations. Populations also varied in their sociability toward conspecifics versus heterospecifics (Fig 3b) and in their level of activity (Fig 3c). Among populations, mean overt aggression was weakly correlated between social contexts (Fig 3a; Pearson's *r* = 0.80, *P* = 0.055) and activity showed a similar trend (Fig 3c; Pearson's *r* = 0.72, *P* = 0.10), while sociability was not correlated (Fig 3b; Pearson's *r* = 0.075, *P* = 0.89).

**Table 4:** Summary of effect tests from a hierarchical MANOVA (PC1–PC3) and separate ANOVA models of each PC, testing variation in brook stickleback (C*ulaea inconstans*) behavior toward conspecifics and heterospecifics (ninespine stickleback, *Pungitius pungitius*). The sympatry/allopatry main effect and its interaction were tested using variation in populations nested in sympatry/allopatry Significant *P*-values are bolded

	Numerator DF	Denominator DF	Wilks' Lambda	*F*-test	*P*-value
MANOVA					
Sym/Allo	3	3	0.01	136.08	**0.001**
Population [Sym/Allo]	12	381	0.88	1.58	0.09
Individuals [Population]	432	427	0.01	3.29	**<0.0001**
Intruder species	3	142	0.81	10.94	**<0.0001**
Intruder species × Sym/Allo	3	142	0.85	0.89	0.45
Intruder species × Population[Sym/Allo]	12	376	0.84	2.06	**0.02**

	Numerator DF	Denominator DF	Mean square	*F*-test	*P*-value
ANOVA PC1					
Sym/Allo	1	4	55.24	9.93	**0.034**
Population [Sym/Allo]	4	144	5.56	1.66	0.16
Individuals [Population]	144	144	3.36	2.95	**0.0006**
Intruder species	1	144	36.91	32.42	**<0.0001**
Intruder species × Sym/Allo	1	4	0.13	0.07	0.8
Intruder species × Population [Sym/Allo]	4	144	1.84	1.61	0.17
Overall error	144		1.14		
ANOVA PC2					
Sym/Allo	1	4	6.38	8.04	**0.047**
Population [Sym/Allo]	4	144	0.79	0.30	0.88
Individuals [Population]	144	144	2.67	3.37	**0.0001**
Intruder species	1	144	0.98	1.24	0.27
Intruder species × Sym/Allo	1	4	0.93	0.44	0.54
Intruder species × Population [Sym/Allo]	4	144	2.10	2.65	**0.035**
Overall error	144		0.79		
ANOVA PC3					
Sym/Allo	1	4	1.18	0.24	0.65
Population [Sym/Allo]	4	144	4.93	2.72	**0.032**
Individuals [Population]	144	144	1.81	3.66	**<0.0001**
Intruder species	1	144	0.41	0.83	0.36
Intruder species × Sym/Allo	1	4	0.67	0.66	0.46
Intruder species × Population [Sym/Allo]	4	144	1.02	2.06	0.089
Overall error	144		0.50		

Rank correlations between conspecific and heterospecific behavior for each population were all positive, and the mean correlations of each behavior averaged among allopatric and among sympatric populations were all significant (Table 5). However, sociability was more strongly correlated between social contexts in sympatric than in allopatric populations (Table 5; Fig 2b): all three sympatric populations had strong correlations (Spearman rank correlation: all *P* < 0.002) while only one allopatric population had a significant correlation (*P* = 0.02). The mean correlation for sociability across the three sympatric populations barely overlapped the confidence interval for the mean allopatric correlation. In contrast, the mean correlations for overt aggression and activity were very similar between allopatric and sympatric populations.

**Table 5:** Number of individuals with valid conspecific and heterospecific trials (*n*), Spearman rank correlation coefficients, and variance estimates (in round parentheses: conspecific, heterospecific) for brook stickleback (C*ulaea inconstans*) behavior toward conspecific and heterospecific intruders (ninespine stickleback, *Pungitius pungitius*)

Lake	*n*	Overt aggression (PC1)	Sociability (PC2)	Activity (PC3)
Allopatric populations				
Armitage	22	0.698*** (2.19, 2.06)	0.388 (1.10, 1.37)	0.743*** (2.36, 2.24)
Bea	27	0.662*** (2.72, 3.08)	0.502* (1.51, 1.64)	0.511* (0.77, 0.76)
Dewhirst	19	0.215 (2.29, 1.67)	0.442 (1.24, 1.91)	0.322 (1.10, 0.86)
Mean correlation and 95% CI		0.53*** [0.22–0.83]	0.44* [0.38–0.51]	0.53*** [0.29–0.76]
Sympatric populations				
Garrison	23	0.409* (2.36, 1.47) *	0.734*** (1.89, 2.01)	0.662** (0.73, 1.06)
Lallan	41	0.551*** (2.06, 1.81)	0.502*** (1.88, 1.71)	0.546** (1.14, 1.48)
Rozon	18	0.250 (3.16, 2.58)	0.646** (1.92, 2.68)	0.316 (0.16, 0.82) ***
Mean correlation and 95% CI		0.40*** [0.23–0.57]	0.63*** [0.50–0.76]	0.51*** [0.31–0.71]

* *P* < 0.05;

** *P* < 0.01;

*** *P* < 0.001.

Among populations, there was only weak evidence that variances in conspecific sociability were lower in allopatric compared with sympatric populations (*P* = 0.08; heterospecific sociability *P* = 0.19). There was also some evidence that the variance for conspecific sociability was lower in populations where conspecific and heterospecific sociability was more weakly correlated (Armitage and Dewhirst) than in the four populations where sociability was more strongly correlated (*P* = 0.055; heterospecific sociability *P* = 0.25). Variance in overt aggression and activity did not differ between allopatric and sympatric populations (all *P* > 0.38) or between populations with and without significant correlations (all *P* > 0.25). Within populations, the variability of behavior differed between conspecific and heterospecific contexts in two populations (all other populations *P* > 0.11): overt aggression toward conspecifics was more variable than toward heterospecifics in Garrison, and activity toward heterospecifics was more variable than toward conspecifics in Rozon (Table 5).

### Multiyear variation in HA

For the multiyear PCA, we focus only on PC1 which captured 33% of the variation and reflected overt aggression. Positive scores reflected more bites, bouts, threats, number of times oriented toward intruder, total time oriented away from intruder, and shorter latencies. Mean HA was greater in allopatric populations in 2008 than in 2004 while sympatric populations did not change their level of HA (nested ANOVA [analysis of variance] model, year X sympatry/allopatry: F_1,4_ = 8.54, *P* = 0.04; allopatry: t_136_ = 1.93, *P* = 0.055; sympatry: t_150_ = 1.25, *P* = 0.22).

## Discussion

We evaluated the covariation of behaviors involved in interactions with conspecifics and with heterospecifics among populations of brook stickleback with and without a competitor in order to explore how covariation may influence the expression and evolution of behavior. Our focus on aggressive behavior reflects our interest in testing the idea that there is a positive relationship between conspecific and heterospecific aggression, as is widely assumed in the literature (Peiman and Robinson 2010). By parsing behavior into different components (overt aggression, sociability, and activity), we were also able to compare differences in covariation among these components. All behaviors toward conspecifics were positively correlated with the same behaviors toward heterospecifics, although the strength of correlations varied among populations depending on the behavior and whether the population coexists with the heterospecific competitor. We discuss the behavioral response rates from our study before focusing on whether brook stickleback behavior may be under directional or correlational selection, and addressing the possible causes and consequences of behavior that is correlated between social contexts.

### Behavioral response rates

The extent to which individuals that respond to an experimental situation accurately represent the behaviors expressed in the population is always a concern. A major cause of nonresponsiveness here was likely fear or stress because wild-caught brook stickleback were captured, introduced into an artificial environment, and then only given one chance to respond to an enclosed intruder, all in less than 24 h. Thus, our method may have biased our results toward the more aggressive individuals. Our overall response rates were similar to previous studies involving threespine stickleback (Bakker 1986; Bell 2005), but as in all studies of behavior, nonresponsive individuals could represent a distinct behavioral type. To evaluate this, we reanalyzed heterospecific behavior collected in 2004 on brook stickleback from these same populations who were given a chance to respond to a ninespine stickleback intruder over each of four subsequent test days using a similar protocol, except they had 48 h of acclimation (Peiman and Robinson 2007). Individuals that responded on the first test day had shorter latencies to approach the intruder than those that responded on test days 2–4, but otherwise early and late responders did not differ in mean HA. This indicates that while some brook stickleback are less willing to initiate interactions with an intruder, once started they tend to exhibit similar behaviors. Also in that study, 67% of allopatric and 66% of sympatric brook stickleback responded to heterospecifics on the first test day, which accumulated to 95% and 90% response rates, respectively, by the fourth test day. In the current study, response rates to heterospecifics of 72% in allopatric and 47% in sympatric brook stickleback after the shorter acclimation period are above the values predicted by a polynomial function at 24 h of approximately 37% using the 2004 data (and are almost identical to the 38% response rate in the Rozon fish from the current study). This suggests that more fish would have responded with a longer acclimation to the experimental conditions. Thus, we provisionally conclude that there is little evidence that the nonrespondents excluded from this study exhibit different behaviors from those that were included.

### Selection on behavior

Convergent traits in replicate populations under similar ecological conditions are often taken as indirect evidence of selection (Dingemanse et al. 2007, 2010). We have evidence of consistent differences in the means and patterns of covariation in behavior of brook stickleback from populations sympatric with versus allopatric from ninespine stickleback. The means of both aggression and sociability in sympatric populations were lower than those in allopatric populations. Sociability toward conspecifics covaried more strongly with sociability toward heterospecifics in sympatric compared with allopatric populations and the variance in conspecific sociability was higher in sympatric compared with allopatric populations. These results are consistent with correlational selection acting in sympatry, as it should maintain greater trait variation than directional or stabilizing selection. Collectively, this suggests that selection on behavior is diversifying among populations of brook stickleback sympatric with, versus those allopatric from, ninespine stickleback. Competition with ninespine stickleback favors diversifying selection on trophically related morphological traits in the same populations of brook stickleback (Gray and Robinson 2002), and so may also generate selection on behavior, although this remains to be directly tested.

While selection appears to be diversifying on behaviors between sympatric and allopatric brook stickleback populations, it also appears that correlational selection may be acting on behaviors in sympatry. If behavioral type influences fitness (for reviews see Smith and Blumstein 2008; Dingemanse and Reale 2005), then the strength of behavioral correlations should vary among populations under different ecological conditions (Bell 2005). For example, variation in phenotypic behavioral syndromes has been observed among lab-reared and wild strains of zebra fish (*Danio rerio*) (Moretz et al. 2007) and in threespine stickleback (*Gasterosteus aculeatus*) under high and low predation risk (Bell 2005; Dingemanse et al. 2007, 2010). However, there are only a few studies that have estimated the fitness of behavioral types in populations expressing syndromes (Sih and Watters 2005; Logue et al. 2009; Smith and Blumstein 2010). If a sociability syndrome is adaptive in our sympatric brook stickleback populations, then the fitness of individuals expressing low levels of sociability toward ninespines and conspecifics should be similar to those expressing high levels of sociability toward intruders of both species. Thus, while our results are consistent with correlational selection acting on sociability in sympatric populations, more direct tests of fitness effects are required before we can make this conclusion.

Sociability syndromes are rarely considered in the literature yet may be fairly common (e.g., Huntingford 1976a; Cote et al. 2010) and can also reflect heritable variation (Wright et al. 2003). A sociability syndrome may arise if more social personality types benefit from information gained about the outcomes of interactions with intruders of both species, while less social personality types reduce costs by interacting less with both conspecifics and heterospecifics. However, behavioral syndromes can also arise by other means. For example, the physical condition of an individual may be related to its position on a behavioral continuum, similar to where bolder individuals can be in better physical condition than shy individuals (Caro 1995; Godin and Davis 1995; Milinski and Bolthauser 1995). Behavioral syndromes may also arise in a single generation as a result of strong correlational selection removing individuals with certain behavioral combinations and/or by learning if individuals change their behavior (Bell and Sih 2007). At this point, we know little about the functional and fitness consequences of sociability between ninespine and brook stickleback, nor whether this syndrome reflects learned or evolutionary responses.

We were surprised to find evidence for a sociability but not an aggression syndrome. An adaptive aggression syndrome might be favored when interference competition for benthic resources is strong both among brook stickleback and between brook and ninespine sticklebacks. Under these competitive conditions, phenotypes that are aggressive toward conspecifics and heterospecifics may have enhanced control of resources in territories, while nonaggressive phenotypes may access resources by employing a sneaker strategy (Dubois et al. 2004). This requires that individuals with either high or low aggression toward both species have similar fitness, and so may collapse as the fitness differential between aggressive types increases and selection switches from being correlational to directional. One reason this switch may occur is if resource abundance changes in sympatry in a way that favors one aggressive type over another.

### Covariation of behavior between social contexts

A variety of mechanisms can cause behaviors to covary, which makes inferences about the genetic architecture of behavior based on phenotypic results challenging. Behaviors can covary for nonadaptive reasons when they are regulated by common expression mechanisms (Vowles and Harwood 1966; Huntingford 1976b; Riechert and Hedrick 1993; Bakker 1994; Reale et al. 2007; Pellegrini et al. 2010). The extent to which shared regulatory pathways and expression mechanisms are inherited would reflect additive genetic covariance effects. Nonadditive genetic effects are also possible, and reflect how aspects of the nonheritable background genotype or dominance effects influence the joint expression of behavior. Both additive and nonadditive genetic effects would tend to generate positive correlations between behaviors toward conspecifics and toward heterospecifics.

Here, we use allopatric populations in a preliminary evaluation of the strength of these genetic mechanisms because allopatric brook stickleback and their ancestors are naive with respect to ninespine stickleback. Correlational selection by definition cannot act on stickleback from allopatric populations, and there are no environmental or learned effects which can account for correlated behavior. We found that most behaviors covaried between social contexts in the allopatric brook stickleback from Armitage and Bea lakes. This likely represents some form of common genetic control of behavior, resulting in the level of behavior that is expressed in interactions with conspecifics also being expressed when faced with this heterospecific even though it has never been encountered before. Three observations are consistent with this hypothesis. First, variation in behavior expressed by allopatric brook stickleback toward novel ninespine stickleback can only reflect additive or nonadditive genetic effects because mechanisms that depend on prior interactions with ninespine stickleback are not possible in allopatric fish. Second, all populations exhibited positive correlations for each behavior even though these populations experience different local conditions. Third, the correlated change in mean overt aggression (and the same trend in activity) among populations suggests that behavior expressed toward conspecifics and toward heterospecifics is related (Bell 2005; Dochtermann 2011). Individuals are also known to vary their level of CA in response to population density, predation risk, and familiarity and relatedness to the social partner (Gaudreault et al. 1986; Quinn 1998; Thanh et al. 2005; Bell and Sih 2007; Griffen and Williamson 2008). However, the consistency of these positive relationships in populations facing different environments is difficult to explain except in light of some degree of additive or nonadditive genetic effects on behavior.

In sympatric populations, behavior may also covary due to interactions among individuals within a population. Indirect genetic effects result from the interaction of an individual's genes with those of its social partners and so can depend on partner genotype (Moore et al. 1997; Meffert et al. 2002) and influence the evolution of behavior (Wolf et al. 1999; Wolf 2003; McGlothlin et al. 2010; Wolf and Moore 2010). A heterogeneous social environment can also cause individuals to pick their environment and social niche, which may in turn influence behavior (Formica et al. 2004; Formica and Tuttle 2009; Saltz and Foley 2011). Studies of indirect genetic effects to date have focused on conspecific interactions where the social partner's genotype may be similar or different from a focal individual's, but theoretically indirect effects may also influence behavior toward heterospecifics, such as between closely versus more distantly related species (Peiman and Robinson 2010). Learning is another form of nonadditive genetic effect that could jointly affect behaviors in different situations. For example, communication during social interactions with brook stickleback may differ from interactions with ninespine stickleback because of differences in the signaling repertoire of each species. We hypothesize that social effects are more likely to decouple CA from HA even if they are under common genetic control because the different genotypes of the social partners are an additional random factor influencing behavior in each interaction. Therefore, social effects provide an unlikely explanation for the stronger correlations observed for sociability in sympatric compared with allopatric stickleback.

Theory suggests that phenotypic correlations may in general estimate genetic correlations for heritable traits (Lande 1979) and heritable variation is present for CA and HA in stickleback (Bakker 1986, 1994; Peiman and Robinson 2007). However, the phenotypic correlations expressed in fish from allopatric populations at best represent an upper limit on genetic covariation because while they cannot be inflated by shared environmental effects, they may still be inflated by nonadditive genetic effects or nonrandom mortality. Evaluating the true genetic covariance of aggression between conspecific and heterospecific contexts will require more direct quantitative genetic methods (Dochtermann and Roff 2010).

Any explanation of why aggressive behavior (and activity) was not correlated between social contexts in the allopatric Dewhirst and sympatric Rozon populations must invoke unique features of these populations relative to the others. A simple explanation may be reduced statistical power resulting from low variance or sample size. There was no evidence that variation in aggression or activity was lower for either of these populations compared to the four where correlations were detected, nor that variation in behaviors differed between conspecific and heterospecific contexts within either population. These two populations had the smallest sample sizes; nonetheless, we detected a significant correlation for sociability in Rozon fish and significant correlations for both aggression and activity were present with only slightly larger sample sizes in other populations. Thus, reduced statistical power is not a compelling explanation. Alternatively, unique social effects may have decoupled HA from CA as discussed above, although this can only occur in sympatric Rozon, and it raises a new uncertainty as to why these effects may be stronger in brook stickleback from Rozon compared with other sympatric populations.

The genetic covariation of HA with other behaviors may provide one mechanism for the unexpected increase in HA in allopatric brook stickleback from 2004 to 2008. Peiman and Robinson (2007) found that mean HA was greater in sympatric compared to allopatric fish in 2004 but this pattern has reversed because of increased HA in allopatric fish in this study. Heterospecific aggression is functionally neutral in allopatry and so it should drift independently among populations rather than consistently increase. Genetic covariation between HA and CA (or some other trait) could cause HA to increase if the correlated trait was under selection and increased in allopatric populations. Unfortunately, we cannot evaluate this hypothesis further because CA was not assessed in 2004.

It seems reasonable that the covariation of the same behaviors in different situations will be stronger than the covariation of different behaviors in different situations (Sih et al. 2004b), but this prediction has rarely been tested. Either type of covariation may be adaptive if generated under correlational selection, but shared genetic mechanisms should be more likely in the former case (Vowles and Harwood 1966; Huntingford 1976b; Riechert and Hedrick 1993; Bakker 1994; Reale et al. 2007; Pellegrini et al. 2010). Most studies of behavioral syndromes, however, focus on correlations between different behaviors and situations (Sih et al. 2004a,b) which should result in weaker correlations between behaviors because of their flexible nature (Dochtermann 2011) or because of social effects as discussed above. For example, Moretz et al. (2007) studied shoaling, activity level, boldness, feeding latency, and CA in zebra fish (*Danio rerio*) and found no strong evidence that these behaviors were genetically correlated, but did not evaluate correlations between each of these behaviors under different conditions. We found that the same behaviors generally covaried between social contexts, and so suspect that the prevalence of syndromes may increase with more studies conducted on the same behaviors across situations.

The causes and consequences of behavioral variation and covariation are complex and generally poorly understood (Dochtermann 2011). Ours is one of the few studies to compare how the same behaviors covary in different social situations among replicate populations in divergent selective environments. We found that behavior toward conspecifics and heterospecifics was positively correlated. The presence of correlations between conspecific and heterospecific interactions for aggression (and activity) in allopatric populations suggests that these behaviors may be more genetically correlated between social contexts than sociability. Sociability was more strongly correlated in sympatry, suggesting that it may be under correlational selection where brook and ninespine stickleback coexist. This parallels other differences in mean behavior and morphology between sympatric and allopatric populations that suggest that interspecific competition generates diversifying selection in this system. This is the first evidence that interspecific competition may generate correlational selection on social behavior, in addition to diversifying selection on behavior and morphology.
